# Recognizing pathology of renal tumor from macroscopic cross-section image by deep learning

**DOI:** 10.1186/s12938-023-01064-4

**Published:** 2023-01-20

**Authors:** Zefang Lin, Weihong Yang, Wenqiang Zhang, Chao Jiang, Jing Chu, Jing Yang, Xiaoxu Yuan

**Affiliations:** 1 Zhuhai Interventional Medical Center, Zhuhai Precision Medical Center, Zhuhai People’s Hospital, Zhuhai Hospital Affiliated with Jinan University, Jinan University, Zhuhai, China; 2 Department of Urology, Zhuhai People’s Hospital, Zhuhai Hospital Affiliated with Jinan University, Jinan University, Zhuhai, China; 3 Department of Medical Equipment Engineering, Zhuhai People’s Hospital, Zhuhai Hospital Affiliated with Jinan University, Jinan University, Zhuhai, China; 4 Department of Pathology, Zhuhai People’s Hospital, Zhuhai Hospital Affiliated with Jinan University, Jinan University, Zhuhai, China; 5 Nursing Department, Guizhou Aerospace Hospital, Zunyi, China

**Keywords:** Renal tumor, Deep learning, Classification

## Abstract

**Objectives:**

This study aims to develop and evaluate the deep learning-based classification model for recognizing the pathology of renal tumor from macroscopic cross-section image.

**Methods:**

A total of 467 pathology-confirmed patients who received radical nephrectomy or partial nephrectomy were retrospectively enrolled. The experiment of distinguishing malignant and benign renal tumor are conducted followed by performing the multi-subtypes classification models for recognizing four subtypes of benign tumor and four subtypes of malignant tumors, respectively. The classification models used the same backbone networks which are based on the convolutional neural network (CNN), including EfficientNet-B4, ResNet-18, and VGG-16. The performance of the classification models was evaluated by area under the receiver operating characteristic curve (AUC), sensitivity, specificity, and accuracy. Besides, we performed the quantitative comparison among these CNN models.

**Results:**

For the model to differentiate the malignant tumor from the benign tumor, three CNN models all obtained relatively satisfactory performance and the highest AUC was achieved by the ResNet-18 model (AUC = 0.9226). There is not statistically significance between EfficientNet-B4 and ResNet-18 architectures and both of them are significantly statistically better than the VGG-16 model. The micro-averaged AUC, macro-averaged sensitivity, macro-averaged specificity, and micro-averaged accuracy for the VGG-16 model to distinguish the malignant tumor subtypes achieved 0.9398, 0.5774, 0.8660, and 0.7917, respectively. The performance of the EfficientNet-B4 is not better than that of VGG-16 in terms of micro-averaged AUC except for other metrics. For the models to recognize the benign tumor subtypes, the EfficientNet-B4 ranked the best performance, but had no significantly statistical difference with other two models with respect to micro-averaged AUC.

**Conclusions:**

The classification results were relatively satisfactory, which showed the potential for clinical application when analyzing the renal tumor macroscopic cross-section images. Automatically distinguishing the malignant tumor from benign tumor and identifying the subtypes pathology of renal tumor could make the patient-management process more efficient.

## Introduction

The incidence of renal cell carcinoma (RCC) increased steadily, mostly on account of incidental detection via cross-sectional imaging [1, 2]. Partial nephrectomy (PN) has become the gold standard treatment for <4 cm renal masses, as studies demonstrated that PN had similar long-term cancer-specific survival results with radical nephrectomy (RN) [3]. After observing the increased overall survival and oncological efficacy in T1a (<4 cm) tumors, PN can be utilized with long-term disease-free survival and low morbidity in T1b (4–7 cm) tumors [4]. Negative surgical margins (NSM) of the pathological specimen indicate a successful excision of PN, however, positive surgical margin (PSM) rates is not low, varying between 0 and 10.6% after PN [5–8]. Management options for PSM include radical nephrectomy, re-resection of the tumor bed, or observation. Though the oncologic impact of positive surgical margins after PN is still controversial [8–14], every effort must be performed to solve this dilemma.

Intraoperative frozen section (IFS) analysis is used to confirm the pathology during PN. However, surgical margin evaluation using IFS analysis is unreliable and time consuming [15, 16]. Despite reduction in PSM rates in IFS group, this data did not show that FS use could improve recurrence-free survival [17]. If we could differentiate benign tumor from malignant tumor or even recognize the subtypes at the suspicious positive cutting plane endoscopically or from macroscopic cross-section image of renal tumor when taken out of body in operation room instead of time-consuming frozen section analysis, quick decision could be made and re-resection of tumor bed or radical nephrectomy might be spared.

Macroscopic cross-sectional imaging is a low-cost, efficient, and convenient image acquisition method which can be implemented by the mobile phone or digital camera. With the successful application in the fields of nature image processing by automatically extracting texture features, deep learning framework, especially the CNN, have been widely used in medical image analysis to classify disease or lesion types, segment the organs or tumors, detect the lesions, and so on [18–20]. The authors proposed a deep learning-based artificial neural network method to classify the chronic renal disease [21]. Wu et al. proposed a multi-feature fusion CNN architecture to automatically identify the kidney abnormalities when analyzing abdominal ultrasound images [22]. Lin et al. proposed a CNN-based method to segment the retinal vessels [23]. The CNN-based multi-scale cost-sensitive neural networks was proposed to evaluated the lung nodules malignancy [24]. Besides, numerous attempts were made to investigate the automated diagnosis of renal tumor.

Lee et al. developed a deep learning-based feature classification method that the deep features and hand-crafted features were concatenated to distinguish benign angiomyolipoma without visible fat from malignant clear cell RCC [25]. The artificial neural network was used to distinguish non-clear cell RCC from clear cell RCC based on corticomedullary phase CT images [26]. Han et al. developed a deep learning framework to classify three subtypes of RCC using 3-phase CT images [27]. Considering that there is substantial overlap in the imaging findings of benign and malignant renal masses, Coy et al. used both of deep learning and radiomics method to distinguish clear cell RCC from benign oncocytoma based on multiphasic CT images [28]. Kwang-Hyun et al. proposed to identify five major histologic subtypes of renal tumors based on multi-phase CT using the end-to-end deep learning framework [29]. Xi et al. developed a deep learning model to distinguish the benign tumors from RCC based on routine MR imaging [30]. Baghdadi et al. developed and evaluated CNNs model to differentiate CD117(+) oncocytoma from the chromophobe subtype of RCC based on CT imaging [31]. Tanaka et al. used CNN-based Inception-v3 architecture to identify the small renal mass on multi-phase contrast-enhanced CT and performed multivariate logistic regression analysis, concluding that deep CNN model makes it possible differentiating the small solid renal masses in dynamic CT images [32]. Zheng et al. built a novel CNN model to identify the four subtypes of the renal parenchymal tumors in T2-weighted fat saturation sequence magnetic resonance images [33]. With an aim to identify two subtypes of benign renal masses and three subtypes of malignant renal masses, Oberai et al. applied CNN-based deep learning method to multi-phase contrast-enhanced CT images [34]. Similarly, based on contrast-enhanced CT images, Zabihollahy et al. aggregated the prediction results from CNN by using the decision fusion-based model to identify two subtypes of benign renal tumors and three subtypes of malignant renal tumors [35]. The authors used a deep CNN to distinguish clear cell RCC from renal oncocytoma based on MR imaging [36]. Zhao et al. used residual CNN to differentiate low-grade (grade I–II) from high-grade (grade III–IV) in stage I and II RCC with MRI [37]. In order to screen the small-diameter renal tumors, Sassa et al. generated synthetic CECT images using a learned deep neural networks and assessed its quality concordance with the real CECT images [38]. Li et al. proposed a radiomics nomogram to distinguish the renal oncocytoma and chromophobe renal cell carcinoma based on the CT imaging features and patient characteristics [39].

Although the above studies achieved relatively satisfactory results, more detailed subtypes of the renal tumor are desired to be identified to meet the practical clinical need. Besides, considering that the macroscopic cross-sectional imaging method is fast and low-cost, classifying the renal tumors based on the macroscopic cross-section images is significant and promising to be studied. However, to the best of our knowledge, recognizing more detailed subtypes of the renal masses based on the macroscopic cross-section images by using the deep learning technology has not been investigated. Automatically distinguishing the macroscopic cross-section images of the renal masses may make the diagnosis and treatment process more efficient.

Based on the macroscopic cross-section images of renal tumors, this study aimed to develop and evaluate the CNN-based models to automatically differentiate the malignant renal tumor from benign renal tumors and recognize four classes of malignant tumor and four classes of benign tumor. Specifically, the CNN-based models in this study include the EfficientNet-B4 [40], ResNet-18 [41], and VGG-16 [42] which are the prevailing CNN models.

The rest structure of this paper is organized as follows. In “Experiments” section, we provide the experimental implementation and analyze the experimental performance. The “Conclusions” section concludes the paper. The “Materials and method” section presents the dataset and method in detail.

## Experiments

### Experiments setting

The experimental computer has a Windows 2016 operating system running on an Intel(R) Xeon(R) Gold 6234 CPU and a NVIDIA Tesla V100-PCIE-32GB Graphics Processing Unit. The CNN-based classification networks were built based on Pytorch framework. In the training phase, in order to avoid overfitting to some extent, we utilized the transfer learning strategy which is proved to be effective to improve the representative of the network. Namely, we initialized the backbone network with the pre-trained parameters on the ImageNet dataset and then fine-turned it with our dataset. Note that the parameters outside the backbone network are initialized with he_normal [43]. The learning rate of the binary classification model, malignant multi-class classification and benign multi-class classification are initially set as 1e−3, 1e−4, and 1e−3, respectively, and scaled by a decay rate of 0.1 every 30 epochs. The batch size is set as 16. The network is trained for 200 epochs with ADAM optimizer where the decay rate for the first- and second-order moments are set as 0.9 and 0.999, respectively. The cross-entropy loss function is adopted to update the network parameters for all three models. In training the malignant classification model, the weight of the class of other malignant tumors in the cross-entropy loss is set as 3 and other classes are set as 1 to address the class imbalance as possible.

### Performance measurements

For each class c, the following four results can be found: TP, FN, FP (the examples which are falsely predicted as c) and TN (the examples which are truly predicted as other class). For the binary classification, we evaluate the performance of the classification model by AUC, sensitivity (Sn), specificity (Sp), and accuracy (Acc), which were computed as:

1 $$ {\text{Sn}}={\text{TP}}/({\text{TP}}+{\text{FN}}),$$

2 $$ {\text{Sp}}={\text{TN}}/({\text{TN}}+{\text{FP}}), $$

3 $$ {\text{Acc}}=({\text{TP}}+{\text{TN}})/({\text{TP}}+{\text{FP}}+{\text{TN}}+{\text{FN}}).$$

For the multi-class classification, the model performance was reported as its macro-averaged sensitivity (ma-Sn), macro-averaged specificity (ma-Sp), micro-averaged AUC (mi-AUC), and micro-averaged accuracy (mi-Acc). The mi-Acc are calculated as follows:

4 $$ {\text{mi-Acc}} = {\text{TP}}/\left({\text{TP}} + \sum \limits _{n = i}^i {{\text{FP}}_i}\right), $$

where ${{\text{FP}}_i}$ denotes the number of false negatives of the *i*th class negative sample. Due to the imbalance of our dataset, the AUC was considered as the principal evaluation metric.

### Results and discussion

In this subsection, based on the three prevailing CNN architectures, after training the binary classification model, malignant multi-class classification model and benign multi-class classification model, the performances of these models were evaluated with the test cohort. Note that for the clinical scenes where recognizing malignant/benign and subtypes of malignant or benign are required, the evaluation results of the multi-class classification model is accumulated from that of the binary classification model and the final evaluation results could be calculated by multiplying the evaluation results of two steps. For the clinical scenes where it is easy to distinguish the malignant/benign renal tumor for the physician, only the multi-class classification model is applied to recognize the subtypes of malignant or benign and thus only considering the evaluation results of the multi-class classification model. Besides, we compared their performance by using DeLong tests evaluated on AUC and *p* < 0.05 was considered statistically significant.

#### Performance of the binary classification model

**Fig. 1:**
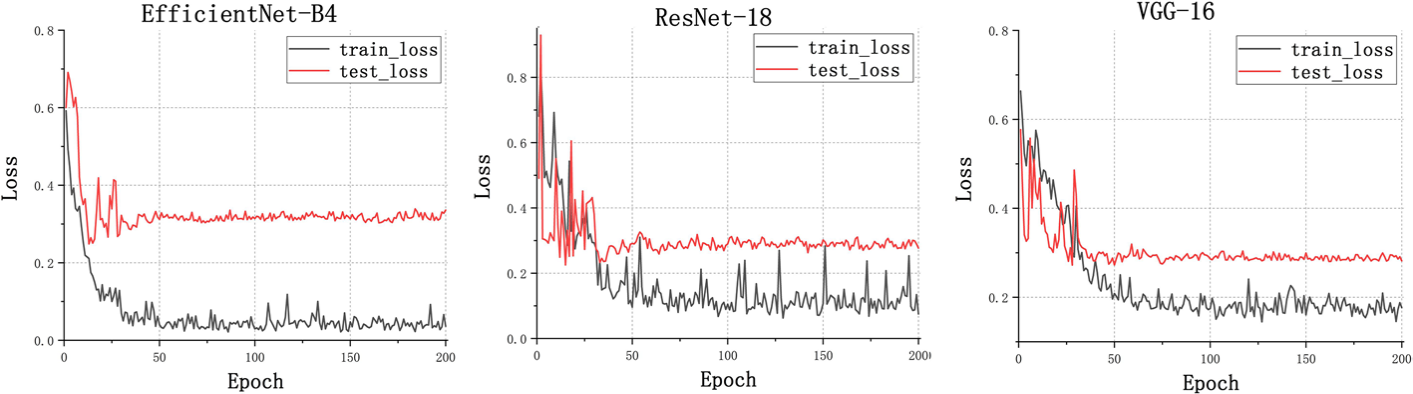
Training loss curve of the binary classification models

**Fig. 2:**
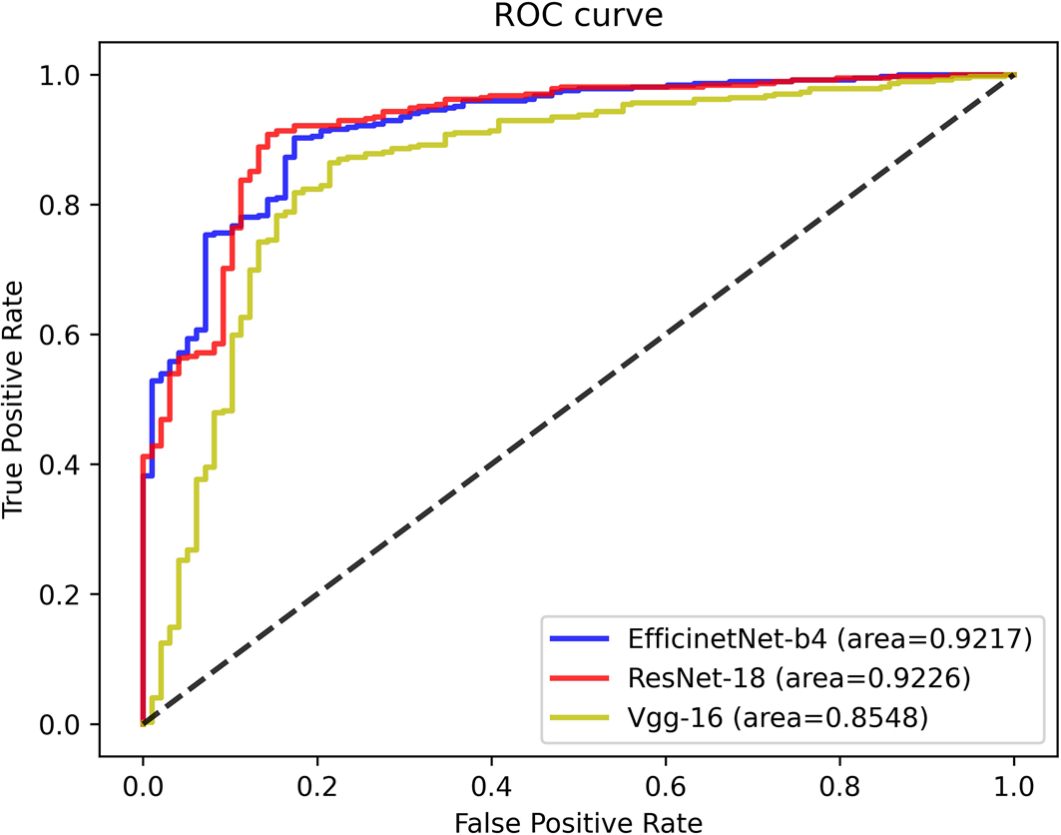
The ROC curve of three models for the binary classification task

**Fig. 3:**
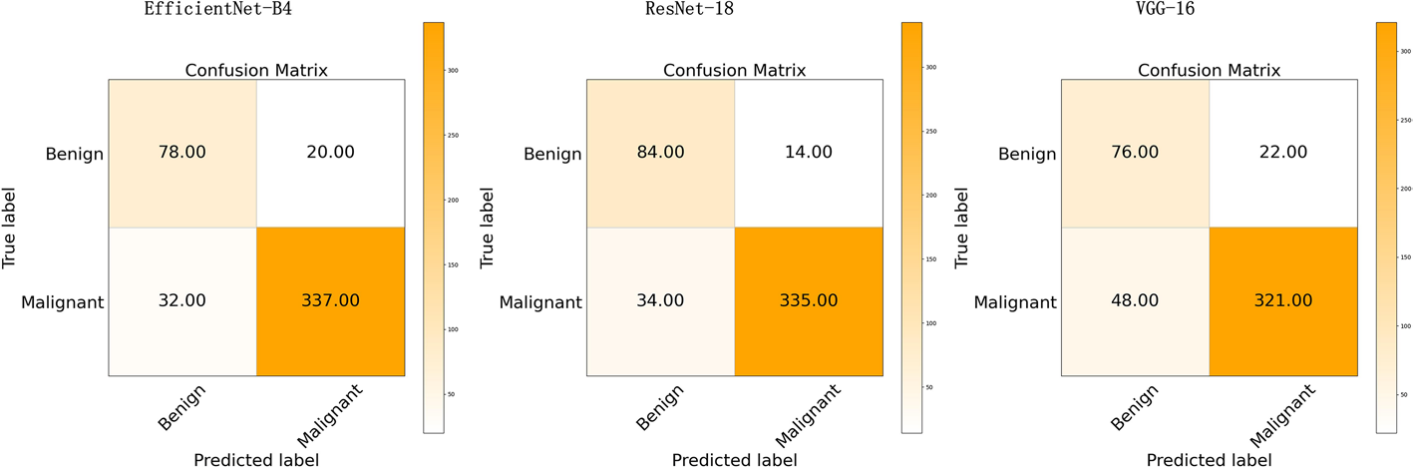
The confusion matrix of three models for the binary classification task

For the binary classification model, Fig. 1 shows the training loss graph for the three CNN models. As shown in this figure, three models converge in the training process near the 50th epoch. The average classification performance of the three CNN architectures is shown in Table 1. Figure 2 presents the receiver operating characteristic (ROC) curve of these models. In general, these models obtained great potential in identifying the malignant tumors from benign tumors (AUC > 0.85). In terms of Sn, the EfficientNet-B4 achieved the best performance. For the performance of AUC, Sp, and Acc, the ResNet-18 ranked the first (0.9226, 0.8572, and 0.8972, respectively). The lowest classification performance was obtained by the VGG-16 network. As Fig. 2 shows, the AUC performance of both of the EfficientNet-B4 and ResNet-18 was statistically significantly higher than that of the VGG-16 model (*p* < 0.05, DeLong test), while there was no significant difference between the AUC of EfficientNet-B4 and ResNet-18 model (*p* > 0.05). For a detailed comparison of the performance and identification of frequent confusion between malignant and benign tumor, the confusion matrix of the binary classification model is illustrated in Fig. 3.

**Table 1:** Predictive performance of binary classification model

CNN models	AUC	Sn	Sp	Acc
EfficientNet-B4	0.9217	**0.9133**	0.7959	0.8887
ResNet-18	**0.9226**	0.9079	**0.8571**	**0.8972**
VGG-16	0.8548	0.8699	0.7755	0.8501

The top results are marked by bold

**Fig. 4:**
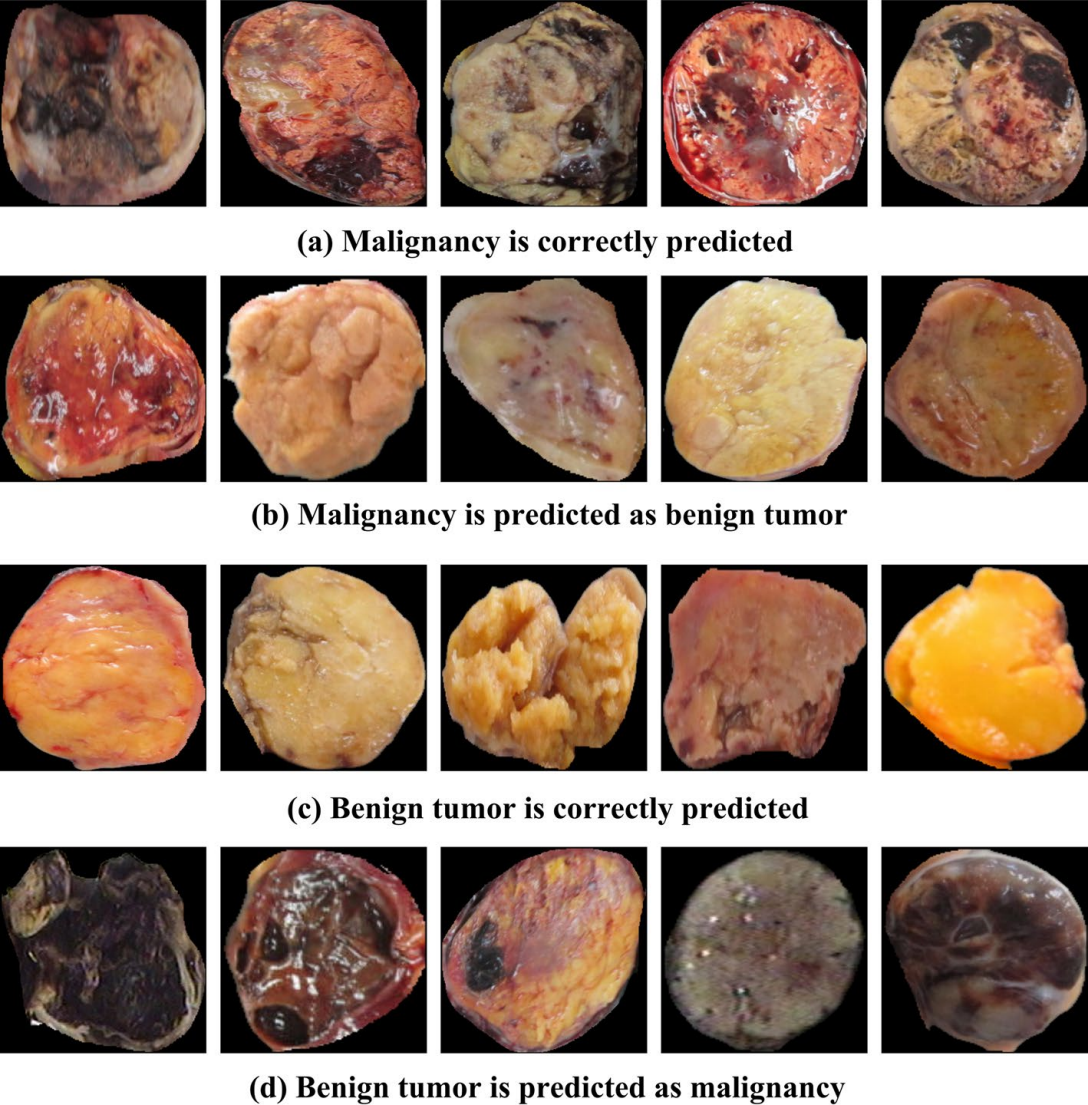
The predicted examples for the binary classification task

As shown in Fig. 4, we randomly selected examples that were misclassified ((b) and (d)) and correctly predicted ((a) and (c)). It can be seen from (b) and (c) that the malignant samples that are predicted as benign tumors are visually similar to most benign tumors and they are lighter and uniform in color. Due to the diversity of the characteristic of the tumor, the texture features from the malignancy are similar to that of the benign tumor and then may make the model difficult to distinguish especially in the situation that they are limited in sample of the data. In contrast to the benign tumor, most malignancies tend to be darker and uneven in color. Thus the benign tumor with the similar features with the malignancy such as darker color and residual blood may be misclassified. This may be because the number of these benign tumors is limited and then the model is not able to learn the distinguishable features well. Furthermore, the reflections on the image potentially influence the judgment of model, which remind us that in the follow-up data collection, the shooting environment can be considered.

As for the binary classification problem, the ResNet-18 and EfficientNet-B4 present a similar and satisfactory performance in distinguishing the malignant renal tumor and benign renal tumor and they had no statistical difference in terms of AUC. On the other hand, the EfficientNet-B4 contains less parameters. Thus the EfficientNet-B4 could be considered the suitable model for renal tumor binary classification task. Furthermore, can be seen from the confusion matrix (Fig. 3), the vast majority of samples can be accurately predicted. The ResNet-18 performs well in distinguishing malignant tumor from benign tumor images with the minimum misclassified samples. Compared to the fact that the benign tumors are predicted to be malignant tumors, the malignant tumors tend to be labeled as benign tumors and the reason maybe that the context features of the malignant tumor is more complex than that of the benign tumor. Besides, the recent studies to differentiate the malignant tumor from the benign tumor were mainly based on the MRI or CT images and their dataset included only one or a few subtypes for malignant tumor and benign tumor, respectively. Lee et al. aimed to differentiate benign angiomyolipoma from malignant clear cell renal cell carcinoma from abdominal contrast-enhanced CT images [25]. Baghdadi et al. performed the binary classification task to identify the benign renal oncocytoma from chromophobe renal cell carcinoma on CT images [31]. Zabihollahy et al. used CNN model to automatically classify RCC and benign tumor based on contrast-enhanced CT images, in which the RCC and benign tumor included three subtypes and two subtypes, respectively [35]. Our binary classification task to identify malignant tumor from benign tumor included 19 kinds of renal tumor subtypes which reflects an unbiased and consecutive dataset, a real disease distribution in clinical practice.

#### Performance of the multi-class classification models

**Fig. 5:**
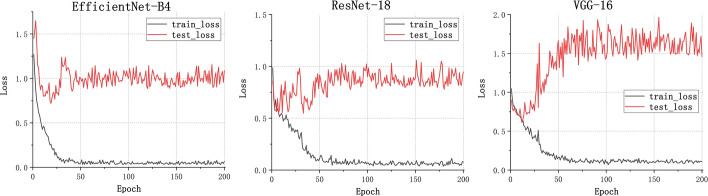
Training loss curve of the malignant multi-class classification models

**Fig. 6:**
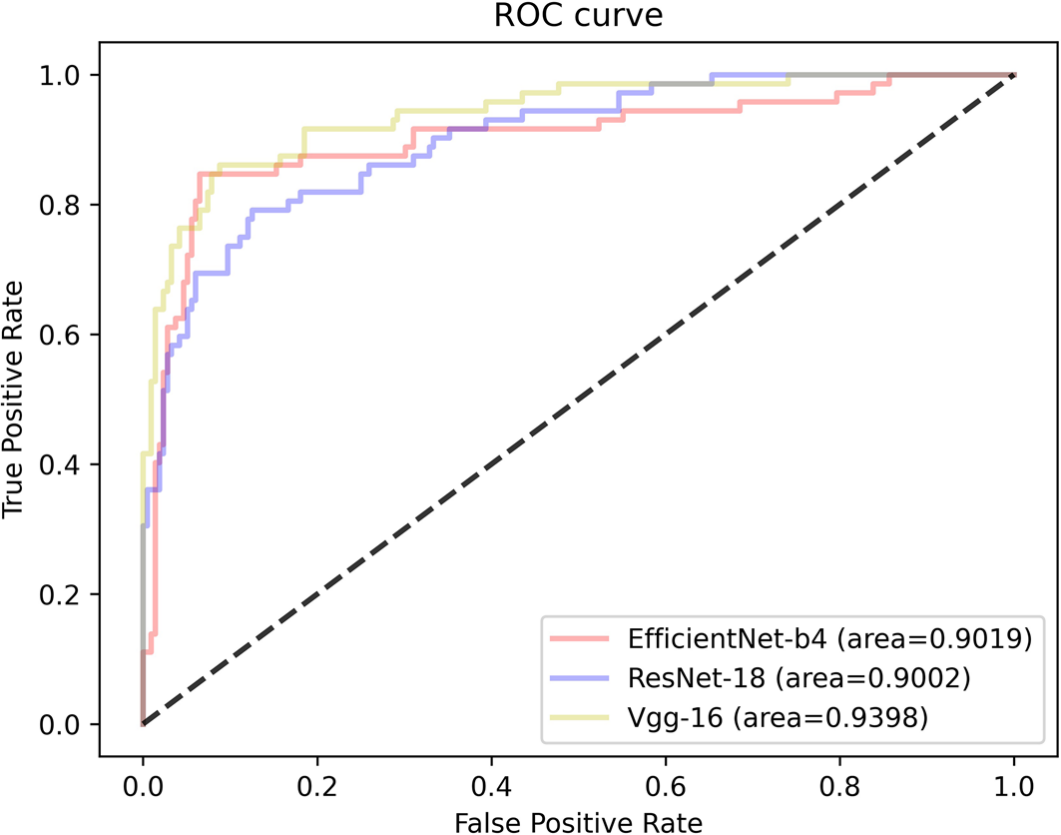
The ROC curve of the malignant multi-class classification model

**Fig. 7:**
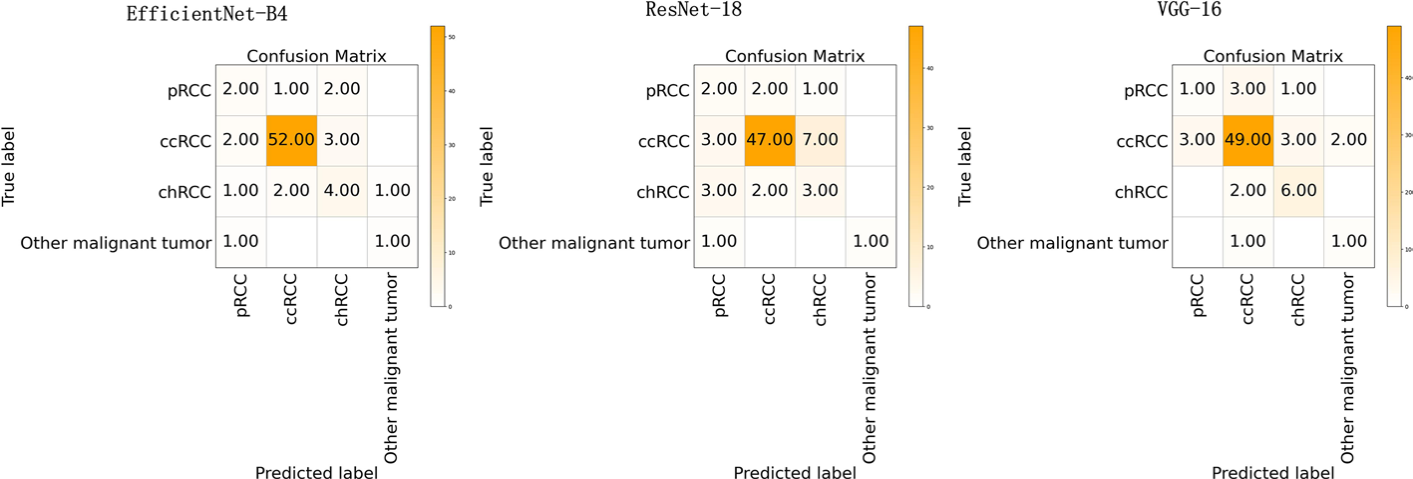
The confusion matrix of the malignant multi-class classification models

For the malignant multi-class classification model, Fig. 5 shows the training loss graph for the three CNN models. The loss of them converge in the training process near the 60th epoch. The average classification results for all the malignant subtypes are shown in Table 2 and Fig. 6. Specifically, the mi-AUC of three CNN models were greater than 0.9 (0.9019, 0.9002, and 0.9398 for EfficientNet-B4, ResNet-18, and VGG-16, respectively). The EfficientNet-B4 achieved the best performance among three models in terms of ma-Sn, ma-Sp, and mi-Acc with the values of 0.5781, 0.9120, and 0.8194. Although the three models obtained relatively satisfactory performance in terms of mi-AUC, ma-Sp, and mi-Acc, their ma-Sn are poor with the values of 0.5781, 0.5249, and 0.5774 for EfficientNet-B4, ResNet-18, and VGG-16, respectively. The ResNet-18 had significant difference from VGG-16 model with regard to mi-AUC (*p* < 0.05). The EfficientNet-B4 had no significant difference from ResNet-18 and VGG-16 architectures (*p* > 0.05). Figure 7 shows the confusion matrix of three CNN models for the malignant multi-class classification task.

**Table 2:** Average predictive performance of each CNN for malignant multi-class classification model

CNN models	mi-AUC	ma-Sn	ma-Sp	mi-Acc
EfficientNet-B4	0.9091	**0.5781**	**0.9120**	**0.8194**
ResNet-18	0.9002	0.5249	0.8760	0.7361
VGG-16	**0.9398**	0.5774	0.8660	0.7917

Bold values indicate the top results

**Fig. 8:**
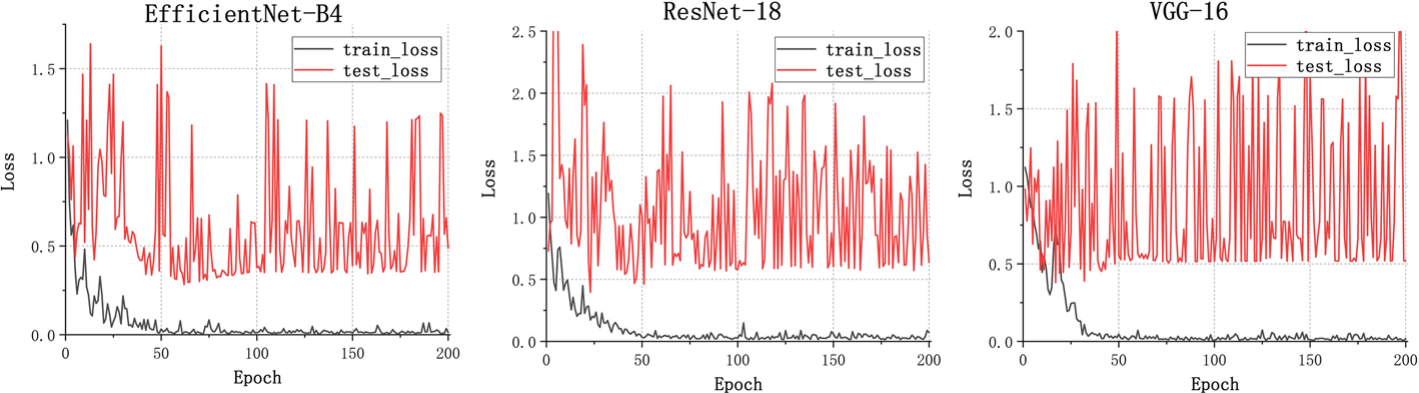
Training loss curve of the benign multi-class classification models

**Fig. 9:**
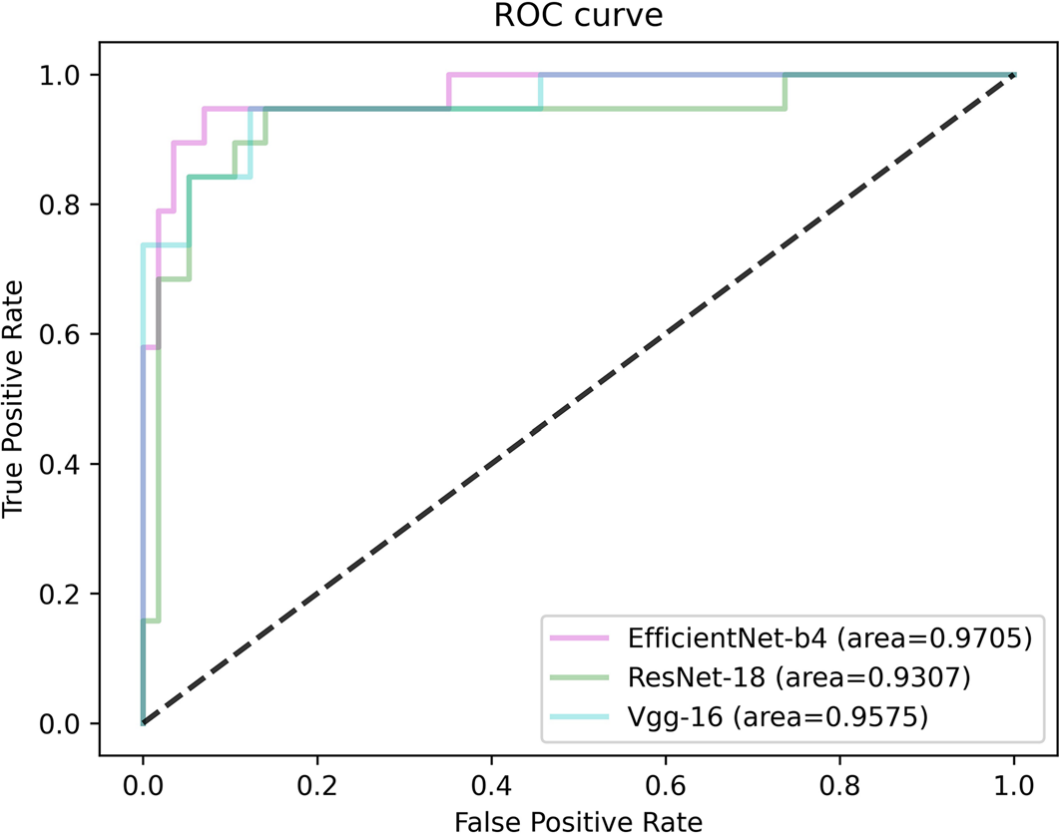
The ROC curve of the benign multi-class classification model

**Fig. 10:**
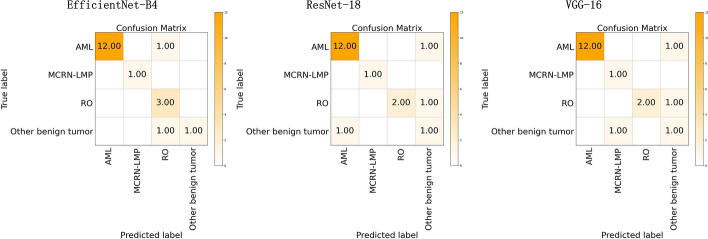
The confusion matrix of the benign multi-class classification models

Figure 8 shows the training loss graph of the three CNN models for the benign renal tumor subtypes classification task. Three models converge in the training process near the 80th epoch. As shown in Table 3 and Fig. 9, for the benign renal tumor subtypes classification model, the mi-AUC of three models obtained relatively satisfactory performance (0.9705, 0.9307, and 0.9575 for EfficientNet-B4, ResNet-18, and VGG-16, respectively) and the mi-Acc, ma-Sn, and ma-Sp of the EfficientNet-B4 ranked the first with the values of 0.8558, 0.9688, and 0.8947 compared to other two models. Specifically, the ma-Sn of the EfficientNet-B4 is about 10% higher than that of the ResNet-18 and VGG-16. However, there was no statistical difference between these models in terms of mi-AUC (*p* > 0.05). Figure 10 shows the confusion matrix of three CNN models for the benign multi-class classification task.

**Table 3:** Average predictive performance of each CNN for benign multi-class classification model

CNN models	mi-AUC	ma-Sn	ma-Sp	mi-Acc
EfficientNet-B4	**0.9705**	**0.8558**	**0.9688**	**0.8947**
ResNet-18	0.9307	0.7724	0.9290	0.8421
VGG-16	0.9575	0.7724	0.9567	0.8421

Bold values indicate the top results

**Fig. 11:**
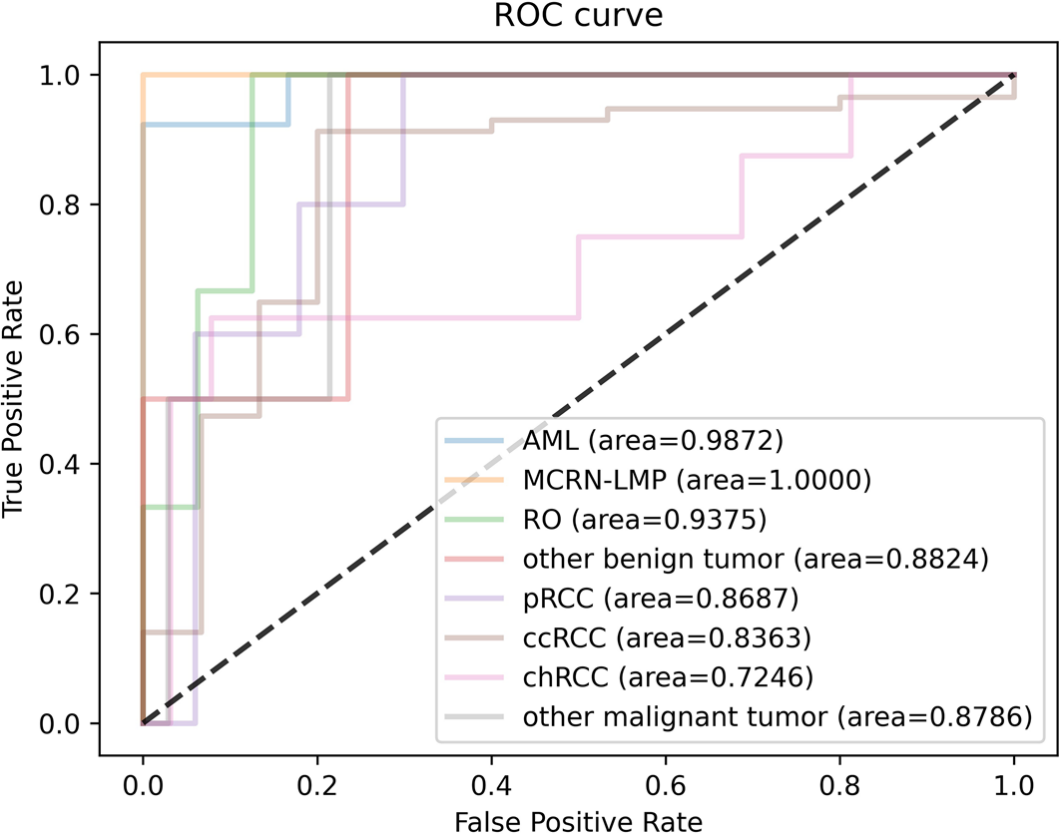
The ROC curve of each class for EfficientNet-B4

**Fig. 12:**
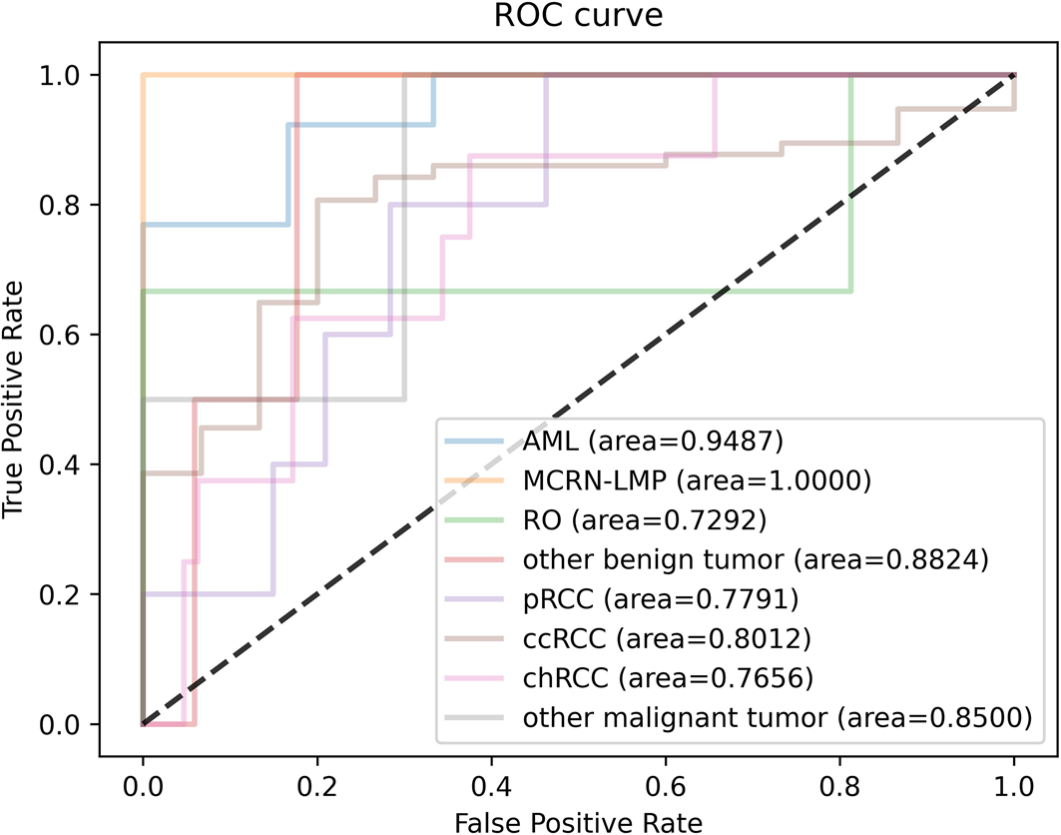
The ROC curve of each class for ResNet-18

**Fig. 13:**
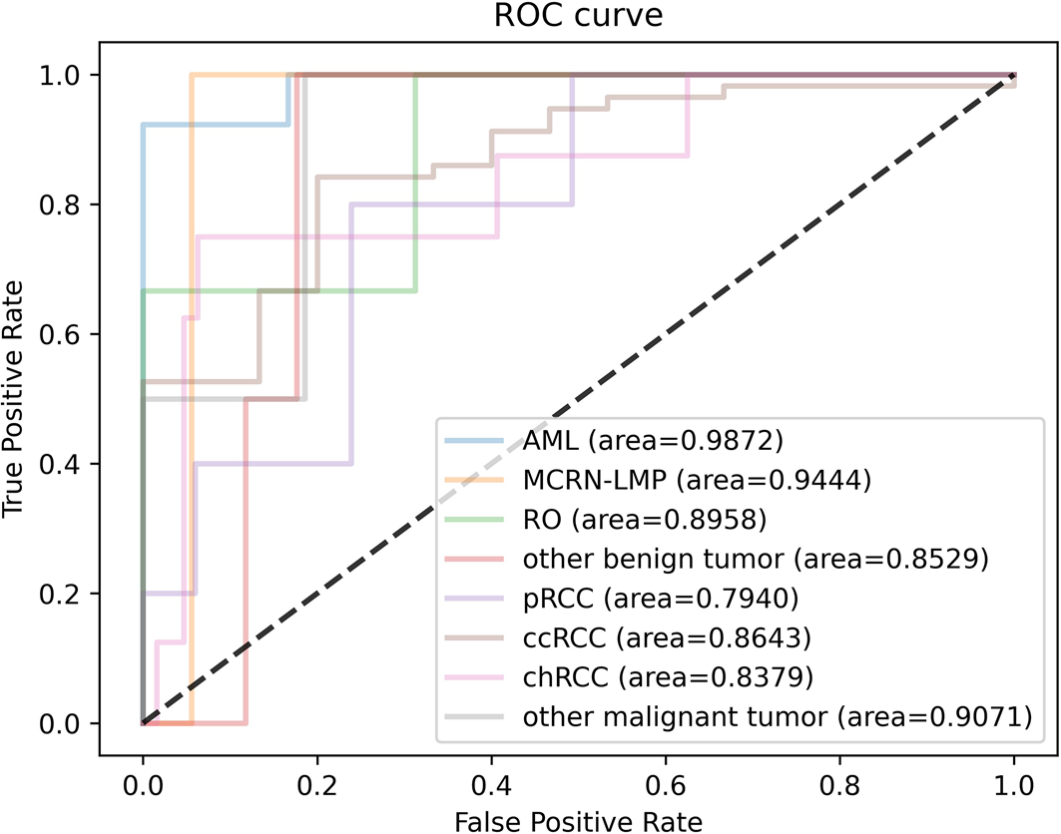
The ROC curve of each class for VGG-16

Specifically, the classification performance of each subtype for three CNN models is shown in Tables 4, 5, and 6, respectively, with the corresponding ROC curve shown in Figs. 11, 12, and 13, respectively. As these tables and figures show, all three models present the potential in renal tumor multi-subtypes classification task. For malignant tumors, the ccRCC with the largest training data achieved the best Sn performance (0.9123, 0.8246, and 0.8596 for EfficientNet-B4, ResNet-18, and VGG-16, respectively) and relatively higher AUC (0.8362, 0.8012, and 0.8642 for EfficientNet-B4, ResNet-18, and VGG-16, respectively). The remaining subtypes with the relatively smaller training data obtained worse Sn performance. More specifically, the Sn of the pRcc are 0.4, 0.4, and 0.2 for EfficientNet-B4, ResNet-18, and VGG-16, respectively. These results may indicate that the imbalance of the data has an effect on the classification performance. For benign tumors, the data amount proportion is relatively balanced than that of malignant tumor. The AML, MCRN-LMP, and RO achieved satisfactory Sn performance for the three models. However, the Sn of the class of other benign tumors for three models is as poor as 0.5 and the reason maybe that this class includes more than more one subtypes and account for a relatively low proportion in dataset, which made the identification more difficult. The overall performance trend of the three CNN models is similar, which proves the consistency from different models when training in the same dataset.

**Table 4:** Predictive performance of each class for EfficientNet-B4 architecture

Metrics	pRCC	ccRCC	chRCC	OMT	AML	MCRN-LMP	RO	OBT
AUC	0.8687	0.8362	0.7246	0.8786	0.9872	1.0	0.9375	0.8824
Sn	0.4	0.9123	0.5	0.5	0.9231	1.0	1.0	0.5
Sp	0.9403	0.8	0.9219	0.9857	1.0	1.0	0.875	1.0
Acc	0.9028	0.8889	0.875	0.9722	0.9474	1.0	0.8947	0.9474

*OMT* other malignant tumors, *OBT* other benign tumors

**Table 5:** Predictive performance of each class for ResNet-18 architecture

Metrics	pRCC	ccRCC	chRCC	OMT	AML	MCRN-LMP	RO	OBT
AUC	0.7791	0.8012	0.7656	0.85	0.9487	1.0	0.7292	0.8824
Sn	0.4	0.8246	0.375	0.5	0.9231	1.0	0.6667	0.5
Sp	0.8955	0.7333	0.875	1.0	0.8333	1.0	1.0	0.8824
Acc	0.8611	0.8056	0.8194	0.9861	0.8947	1.0	0.9474	0.8421

**Table 6:** Predictive performance of each class for VGG-16 architecture

Metrics	pRCC	ccRCC	chRCC	OMT	AML	MCRN-LMP	RO	OBT
AUC	0.7940	0.8643	0.8379	0.9071	0.9872	0.9444	0.8958	0.8529
Sn	0.2	0.8596	0.75	0.5	0.9231	1.0	0.6667	0.5
Sp	0.9552	0.6	0.9375	0.974	1.0	0.9444	1.0	0.8824
Acc	0.9028	0.8056	0.9167	0.9583	0.9474	0.9474	0.9474	0.8421

For the malignant subtypes classification task, although three models obtained relatively satisfactory performance in terms of mi-AUC, their ma-Sn are relatively worse (ma-Sn<0.6). Because some classes obtained worse Sn, like pRCC, chRCC, and other malignant tumors (see Tables 4, 5, and 6), and the one possible explanation is that the similarity of these renal malignant subtypes and the imbalance of the dataset make the extracted features hard to be distinguished. As can be seen from Figs. 6 and 15, the class of pRCC, chRCC, and other malignant tumors account for low training sample size and the confusion matrix of the EfficientNet-B4 model showed that 20 percent of pRCC and 25 percent of chRCC are predicted as chRCC. Similarly, for the other two models, these classes also tend to be predicted as ccRCC. Furthermore, the Sn of the ccRCC achieved the best (Sn > 0.8 for three models) because ccRCC account for largest training sample size (about 78% of training data), and thus the CNN models could learn more representative features about it. For the benign subtypes classification task, the mi-AUC and ma-Sn achieved more than 0.93 and 0.77 for three models. As for the performance of each class, the Sn of the class of other benign tumors is only 0.5 for three models and the reason might be that limited by the amount of the dataset, some subtypes were combined into the class of other benign tumors, which made it more challenging to distinguish. From the confusion matrix of the benign multi-class classification model (see Fig. 10), the MCRN-LMP can be accurately predicted by three models and a small number of samples for other three classes are misclassified, which reveals the potential for CNN model to recognize multi-subtypes renal tumors. From the view of the comprehensive performance of all metrics and the statistical significance in AUC, the EfficientNet-B4 could be considered the best among three models for both of malignant and benign multi-class classification task, which revealed the advantage of EfficientNet-B4 and the certain consistency between two multi-class classification task. Besides, the previously reported studies about renal tumor diagnosis were mainly based on MRI or CT images and covered less subtypes compared with our study [27, 29, 33, 35, 39]. In the clinical practice, more renal tumor subtypes are desired to be diagnosed and the diagnosis process are expected to be as efficient as possible. Our study revealed it is possible to distinguish more subtypes based on the easily available macroscopic cross-section images and established a benchmark for the follow-up studies evaluating the macroscopic cross-section images of renal tumor.

## Conclusions

In this study, we proposed CNN-based method to distinguish malignant renal tumors from benign renal tumors and recognize the multi-subtypes renal tumors. Different from the existing studies that the deep learning technique is used to automatically diagnose the renal tumors based on CT or MRI, we considered the macroscopic cross-section image which are easily available. Besides, in order to adapt to different medical application scenarios, binary classification model and multi-class classification model can be flexibly selected. For the clinical scenes where recognizing malignant/benign and subtype of malignant or benign is required, the binary classification model is firstly used to distinguish the malignant from benign renal tumor to obtain preliminary clinical decision and then the multi-class classification model is adopted to recognize the subtype of renal tumor to make further treatment plan. For the clinical scenes where it is easy to distinguish the malignant/benign renal tumor for the physician, only the multi-class classification model is applied to recognize the subtype of malignant or benign. Besides, since the binary and multi-class classification models require very different features, separating these two steps could make the training of these models easier to some extent especially for the extremely imbalanced data. For the binary classification model and benign multi-class classification model, the experimental results showed that it is considerable to use deep learning method to diagnose the renal tumor. For the malignant multi-class classification model, although the Sn performance for some classes is poor due to the limitation of the data, it supports the possibility of using deep learning for the automated recognition of malignant renal tumor subtypes. As the first solution for diagnosing the renal tumors based on the macroscopic cross-section image, our method demonstrates great potential for future clinical applications.

Although this study had achieved relatively satisfactory diagnostic performance for renal tumor, there are still several limitations which we aim to overcome in the future. First, more data should be collected to improve the prediction performance and generalization of the classification model due to the data-driven nature of deep learning, including increasing the sample size and collecting multi-center patient cohort. Second, external datasets are expected to be considered to further validate our method. Thirdly, due to the limitation of the consecutive sample in this study (resulting in a low proportion of some subtypes), some subtypes were combined into one class. Thus more detailed subtypes of renal tumor will be distinguished after increasing the training samples of each subtype.

## Materials and method

### Data acquisition

We retrospectively reviewed patients who underwent RN and PN for the renal masses in Zhuhai People’s Hospital and Jiangmen Central hospital from January 2015 to December 2020. Macroscopic cross-section image of formalin-fixed mass and postoperative pathology results of each patient were collected. In particular, these macroscopic cross-section images were picked by mobile phone or digital camera and stored in PNG format, which means that macroscopic cross-sectional imaging is a low-cost, efficient, and convenient imaging method. Note that the size of images are 614,768 (from Zhuhai People’s Hospital) and 480,640 (from Jiangmen Central hospital), respectively. 467 cases with a total of 467 renal tumors were included in this study. The exclusion criteria were as follows: (1) cases without macroscopic cross-section images and those with blurry images; (2) carcinoma of renal pelvis who underwent radical nephrectomy or nephro-ureterectomy; (3) cases with renal tumors in children who were under 18.

**Fig. 14:**
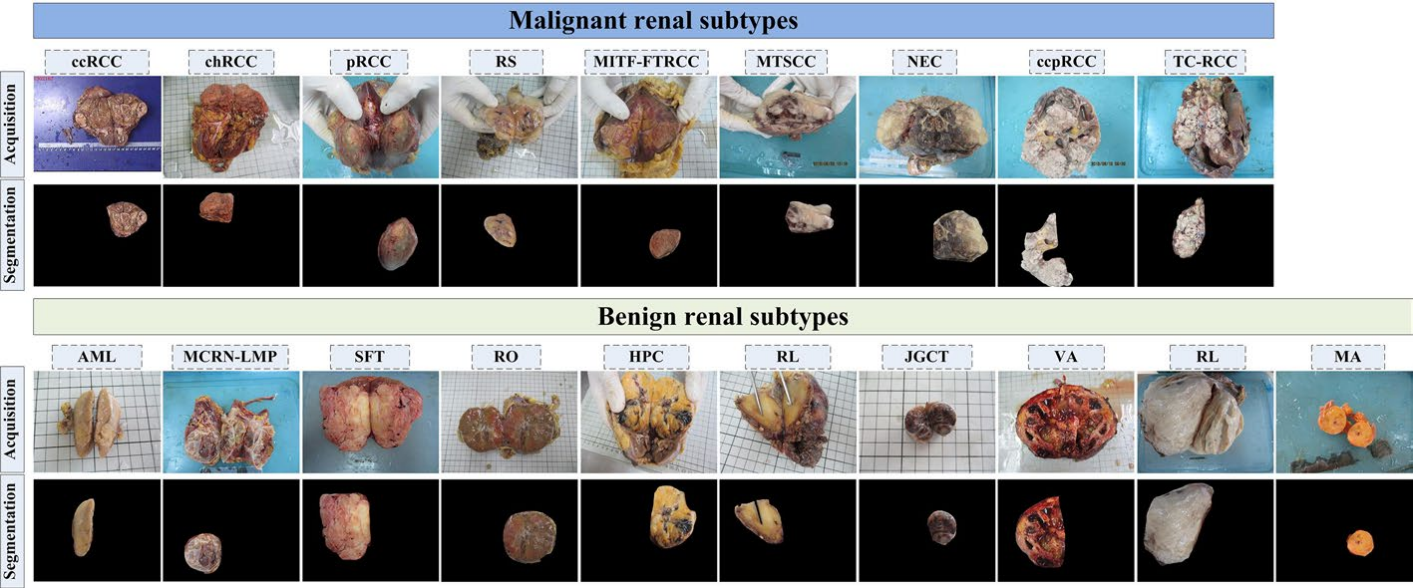
Examples of renal tumor subtypes. (For interpretation of the references to color in this figure legend, the reader is referred to the web version of this article)

Among these tumors, 369 malignant tumors include clear cell renal cell carcinoma (ccRCC), chromophobe renal cell carcinoma (chRCC), papillary renal cell carcinoma (pRCC), renal sarcoma (RS), MiT family translocation renal cell carcinoma (MITF-FTRCC), mucinous tubular and spindle cell carcinoma (MTSCC), neuroendocrine carcinoma (NEC), clear cell papillary renal cell carcinoma (ccpRCC), tubulocystic renal cell carcinoma (TC-RCC), and the remaining 98 benign tumors consist of angiomyolipoma (AML), multilocular cystic renal neoplasm of low malignant potential (MCRN-LMP), solitary fibrous tumor (SFT), renal oncocytoma (RO), hemangiopericytoma (HPC), renal lipoma (RL), juxtaglomerular cell tumor (JGCT), villous adenoma (VA), renal leiomyoma (RL), metanephric adenoma (MA). Table 7 presents the information of each subtype. One senior physician (Xiaoxu Yuan) delineated the regions of interest (ROI) of the tumor using the drawing software (included in the Windows 10 system) and decided which tumor area should be included if there exists more than one in the cross-section image, and then another physician (Wenqiang Zhang) confirmed these delineated tumors. Figure 14 shows the examples of these renal tumor subtypes.

**Table 7:** Description of dataset

Malignant (9 subtypes)										
Subtypes	ccRCC	chRCC	pRCC	RS	MITF-FTRCC	MTSCC	NEC	ccpRCC	TC-RCC	
Sample size (n)	288	42	27	3	2	3	2	1	1	
Benign (10 subtypes)										
Subtypes	AML	MCRN-LMP	SFT	RO	HPC	RL	JGCT	VA	RL	MA
Sample size (n)	66	6	2	16	2	2	1	1	1	11

### Data preprocessing

**Fig. 15:**
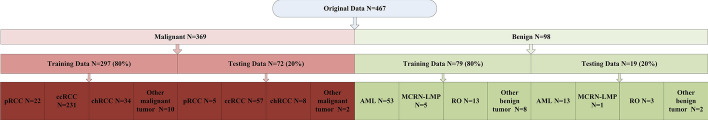
The data cohorts of the multi-class classification task

In the designing of binary classification model, the tumors are identified as malignant tumor or benign tumor. We performed the fivefold cross-validation for this model. For the designing of multi-subtypes classification models, 19 subtypes of renal tumors were recombined to 8 subtypes because the dataset is unbalanced as the number of some subtypes is small. Namely, the MITF-FTRCC, MTSCC, RS, NEC, ccpRCC, and TC-RCC were classified into the class of other malignant tumors. The SFT, HPC, RL, JGCT, VA, RL, and MA were classified into the class of other benign tumors. Ultimately, the classes of malignant tumors include pRCC, ccRCC, chRCC, and other malignant tumors and the classes of benign tumors include RO, AML, MCRN-LMP, and other benign tumors. Note that due to the limitation of the sample size (resulting in a low proportion of some classes), we do not use the cross-validation technique for the multi-class classification models. For the malignant multi-class classification, all the malignant tumors are randomly divided into training data and test data. For the benign multi-class classification, all the benign tumors are randomly divided into training data and test data. Note that these data are divided based on category so as to avoid certain categories from being completely allocated to the training set or the test set. The data cohorts of the multi-class classification task are illustrated in Fig. 15.

**Fig. 16:**
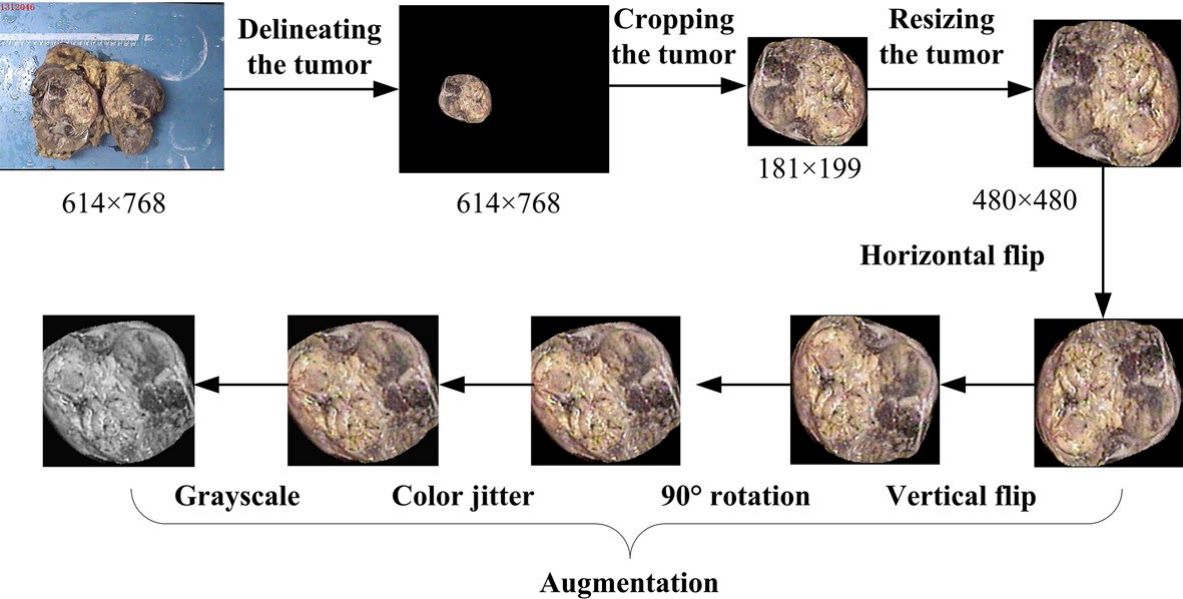
The flowchart of the data preprocessing

Since the ROI accounts for less areas of the image, we cropped the ROI and then resized it into the size of 480 $\times$ 480. The training data were augmented by the operation of random horizontal flip (*p* = 0.4), random vertical flip (*p* = 0.4), 90$^{\circ }$ random rotation, color jitter (contrast = 0.2), and random grayscale (*p* = 0.2) during the model training to avoid overfitting to some extent. The flowchart of the data preprocessing is shown in Fig. 16.

### Classification models

**Fig. 17:**
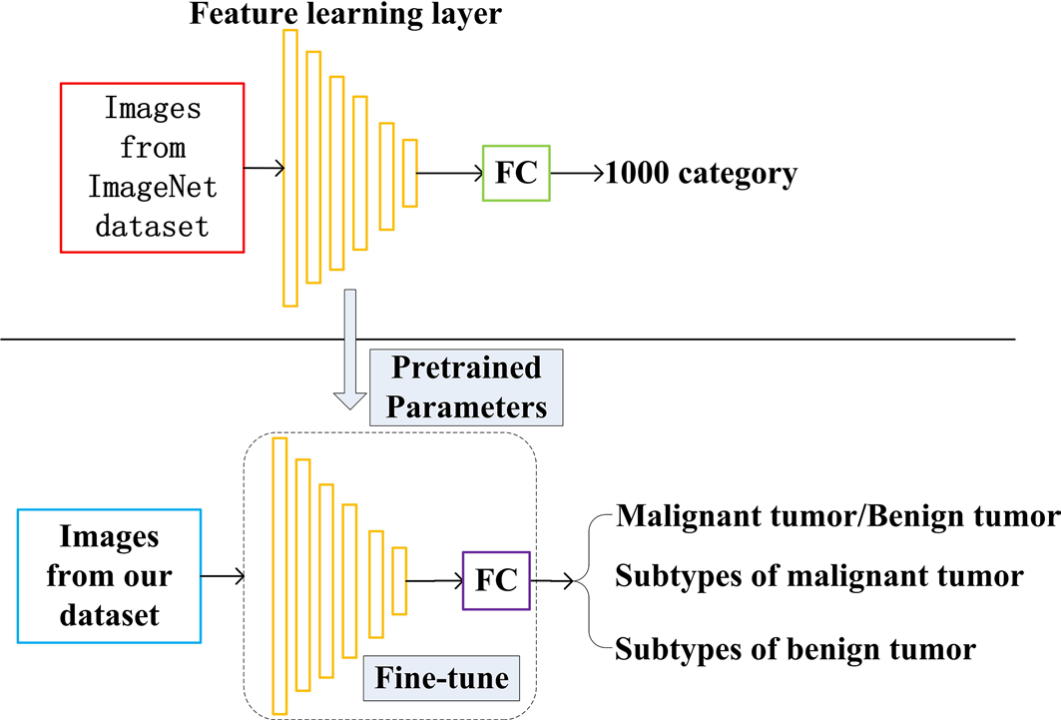
The transfer learning process

In this study, we adopted the EfficientNet-B4, ResNet-18, and VGG-16 as the backbone networks, respectively, for both of the binary and multi-class classification models and these architectures are open source. Specifically, we modified the 1000 category in the last layer of these networks as two category for binary classification, four category for malignant tumor subtypes classification and four category for benign tumor subtypes classification, respectively. These models were pretrained with ImageNet dataset [44] and then fine-tuned with our training dataset with transfer learning method [45]. The transfer learning process is shown in Fig. 17 and the introduction of these architectures are as follows.

#### EfficientNet-B4

**Fig. 18:**
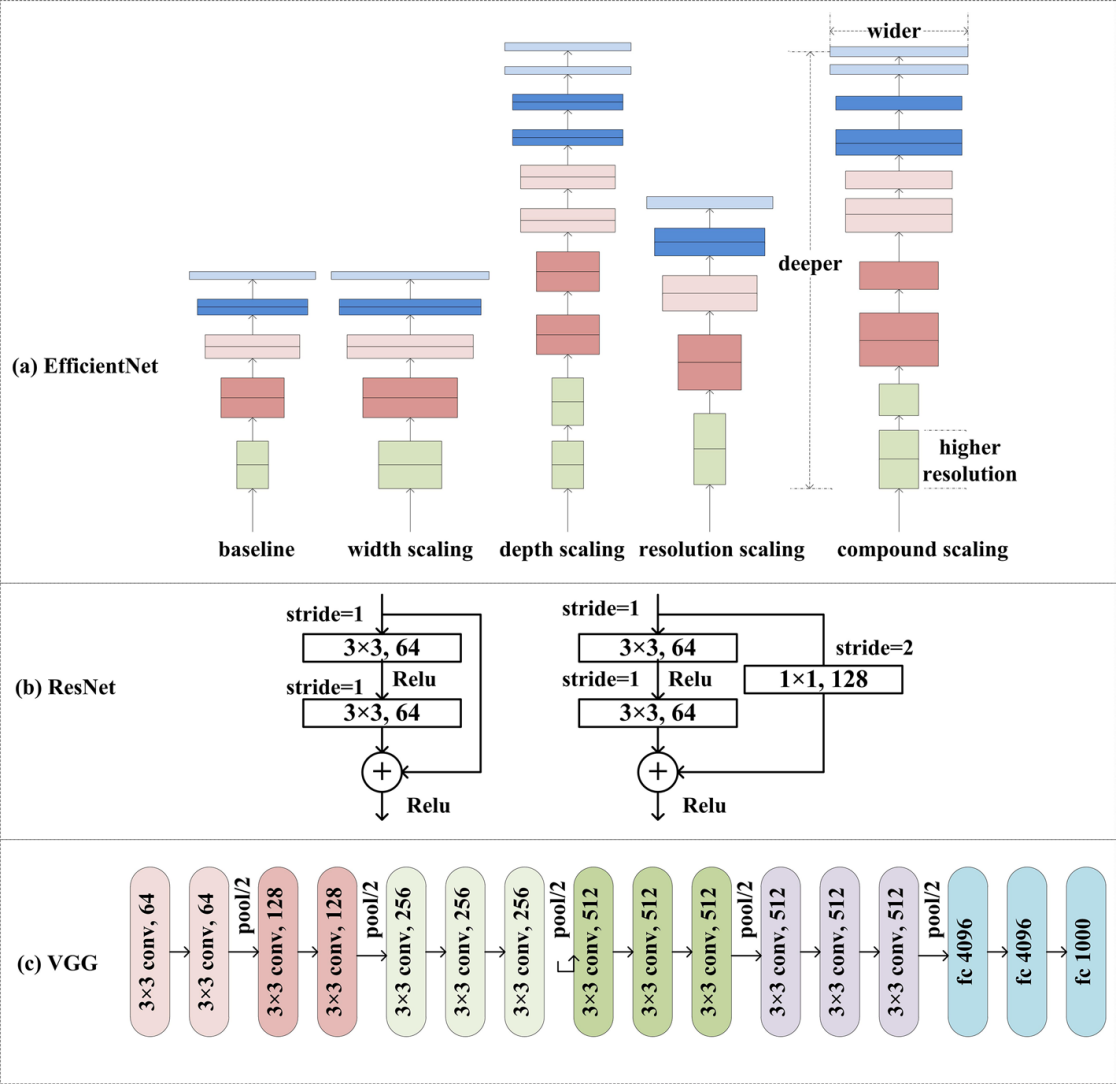
Details for the CNN frameworks

The EfficientNet architecture makes full use of three scaling dimensions (including the width of the network, the depth of the network and the resolution of the input image) to obtain a more suitable network and then to optimize the precision and efficiency of the network. Specifically, a wider network could capture finer-grained features and is easier to train, but extremely wide for the shallow networks making it difficult to capture higher level features. There is a need to coordinate the scales of width and depth. The depth of the network affects the ability of extracting the representative features to some extent. However, when training the network, the deeper network could lead to gradient vanishing and then degenerate the performance. The high resolution of the input image contributes to the capture of more finer-grained pattern [40]. These dimensions are adjusted by a composite coefficient to generate a series of EfficientNet architectures (EfficientNet-B0 to B7) and each architecture has parameters from 5.3 to 66 M. There is a certain relationship between the different dimensions and thus properly coordinating these dimensions is needed for developing a robust network. Among all these architectures, for our classification task, we adopted the EfficientNet-B4 as the backbone of the classification model with the width coefficient, depth coefficient, and input resolution of 1.4, 1.8, and 380. The scaling strategy is shown as (a) in Fig. 18.

#### ResNet-18

The “residual block” was proposed in ResNet architecture to efficiently avoid the gradient vanish to some extent and accelerate the training process and this architecture obtained the champion in the ImageNet Large-Scale Visual Recognition Challenge (ILSVRC) 2015 competition. Specifically, a so-called “short-connection” was implemented between the input and the output of the “residual block” [41]. Here we chose the ResNet-18 as the backbone of our classification task and it contains one general convolutional layer, eight “residual block” modules, one pooling layer, and one full connected layer. Each “residual block” is the stack of two convolutional layers. The “residual block” is shown as (b) in Fig. 18.

#### VGG-16

VGG network was proposed in ILSVRC 2014. It claimed to increase the depth of the network by using very small convolution kernels and consisted of a stack of convolutional layers, maxpooling layer, and full connection layers [42]. In the family of VGG architecture, the VGG-16 was chosen and modified for our task and it has 13 convolutional layers, five pooling layers, and three full connected layers. The network structure diagram is shown as (c) in Fig. 18.

## Data Availability

The datasets used and/or analyzed during the current study are available from the corresponding author on reasonable request.

## Abbreviations

ccRCC: Clear cell renal cell carcinoma
chRCC: Chromophobe renal cell carcinoma
pRCC: Papillary renal cell carcinoma
RS: Renal sarcoma
MITF-FTRCC: MiT family translocation renal cell carcinoma
MTSCC: Mucinous tubular and spindle cell carcinoma
NEC: Neuroendocrine carcinoma
ccpRCC: Clear cell papillary renal cell carcinoma
TC-RCC: Tubulocystic renal cell carcinoma
AML: Angiomyolipoma
MCRN-LMP: Multilocular cystic renal neoplasm of low malignant potential
SFT: Solitary fibrous tumor
RO: Renal oncocytoma
HPC: Hemangiopericytoma
RL: Renal lipoma
JGCT: Juxtaglomerular cell tumor
VA: Villous adenoma
RL: Renal leiomyoma
MA: Metanephric adenoma
